# Supplementary material for: Analysis of chi angle distributions in free amino acids via multiplet fitting of proton scalar couplings
Source: Magn Reson (Gott). 2024 Aug 19;5(2):103–20. doi: 10.5194/mr-5-103-2024 (PMC11570886; doi:10.5194/mr-5-103-2024)
Supplement: The supplement related to this article is available online at: https://doi.org/10.5194/mr-5-103-2024-supplement. [file mr-5-103-2024-supplement.zip › Syed2024/data/fit1d_fitnmr_output.tar.gz!/fit1d/met/500/bmse000044_simulation_1_nmredata/1H/resonances_1d.pdf]

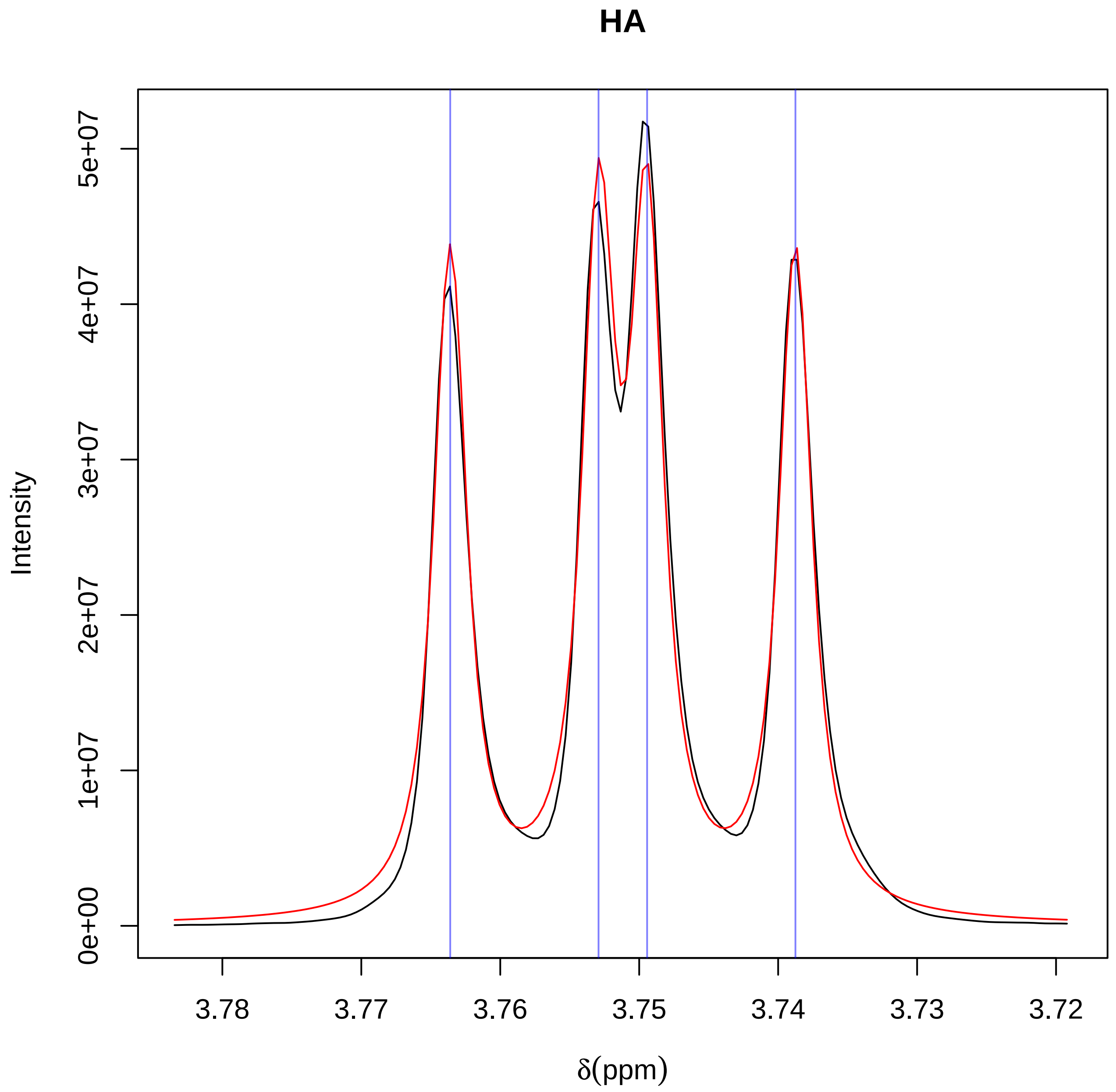

HA
Intensity
0e+00
1e+07
2e+07
3e+07
4e+07
5e+07
3.78
3.77
3.76
3.75
3.74
3.73
3.72
δ(ppm)

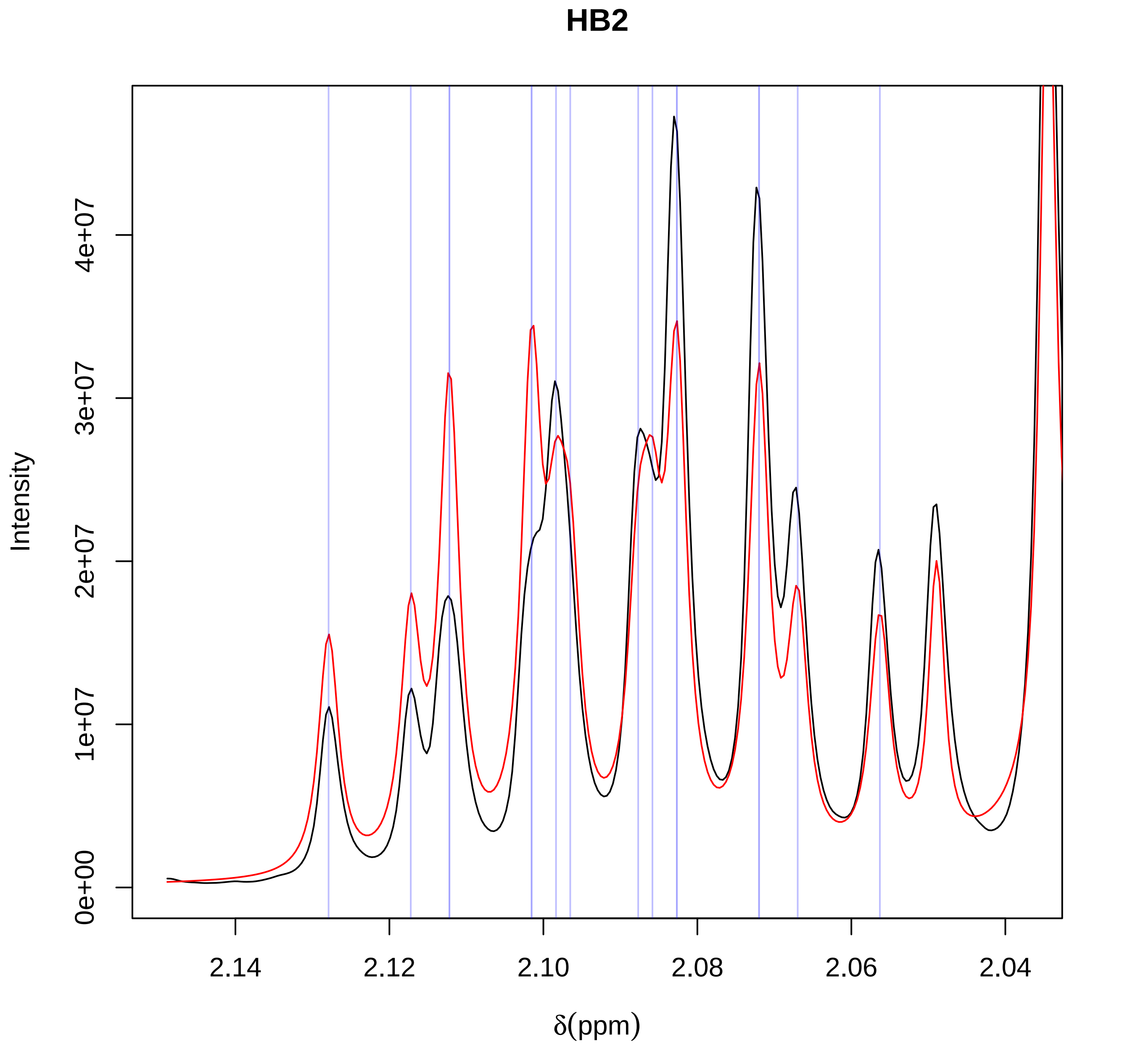

HB2
Intensity
4e+07
3e+07
2e+07
1e+07
0e+00
2.14
2.12
2.10
2.08
2.06
2.04
δ(ppm)

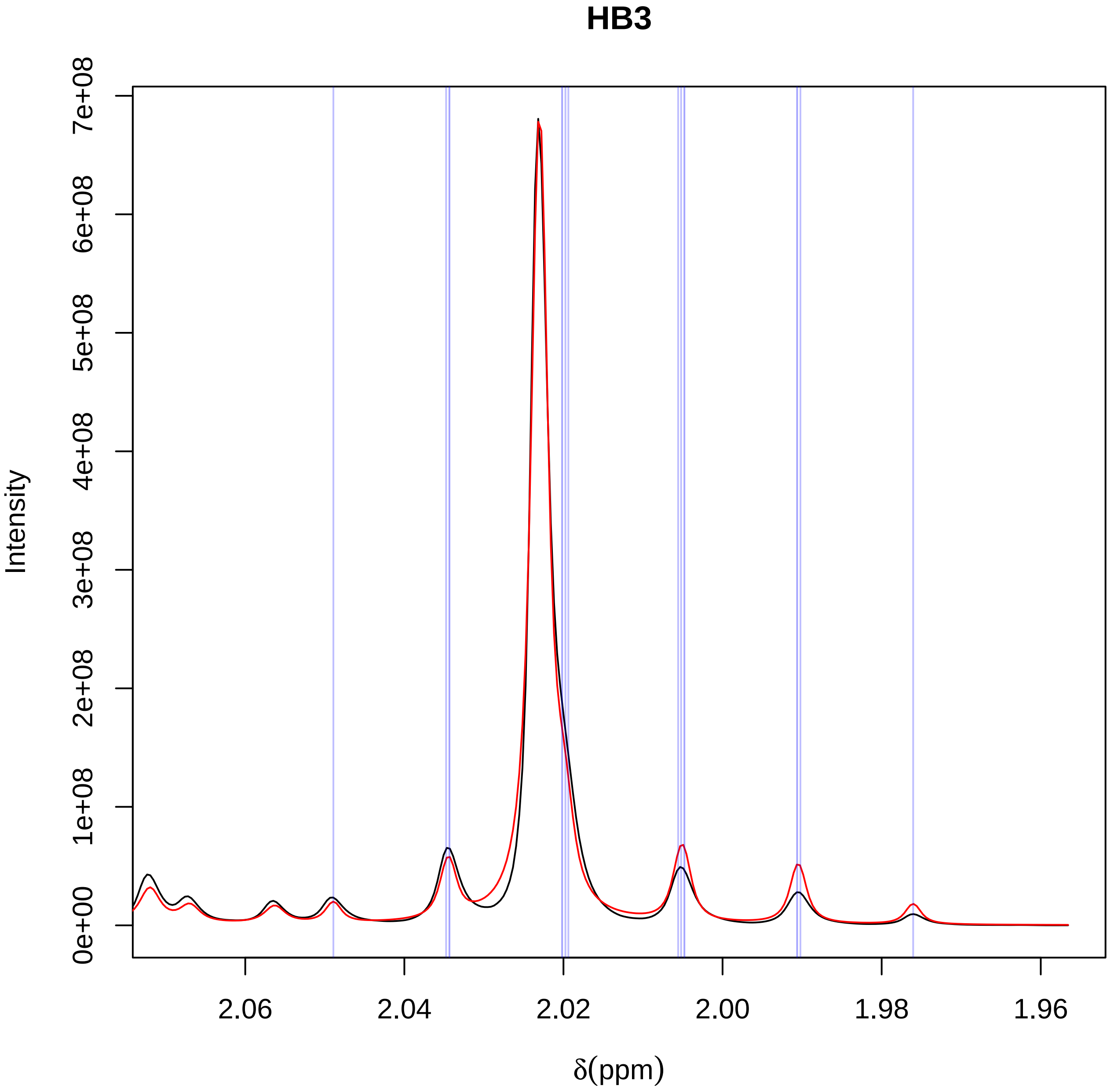
HB3
Intensity
0e+00
1e+08
2e+08
3e+08
4e+08
5e+08
6e+08
7e+08
2.06
2.04
2.02
2.00
1.98
1.96
δ(ppm)

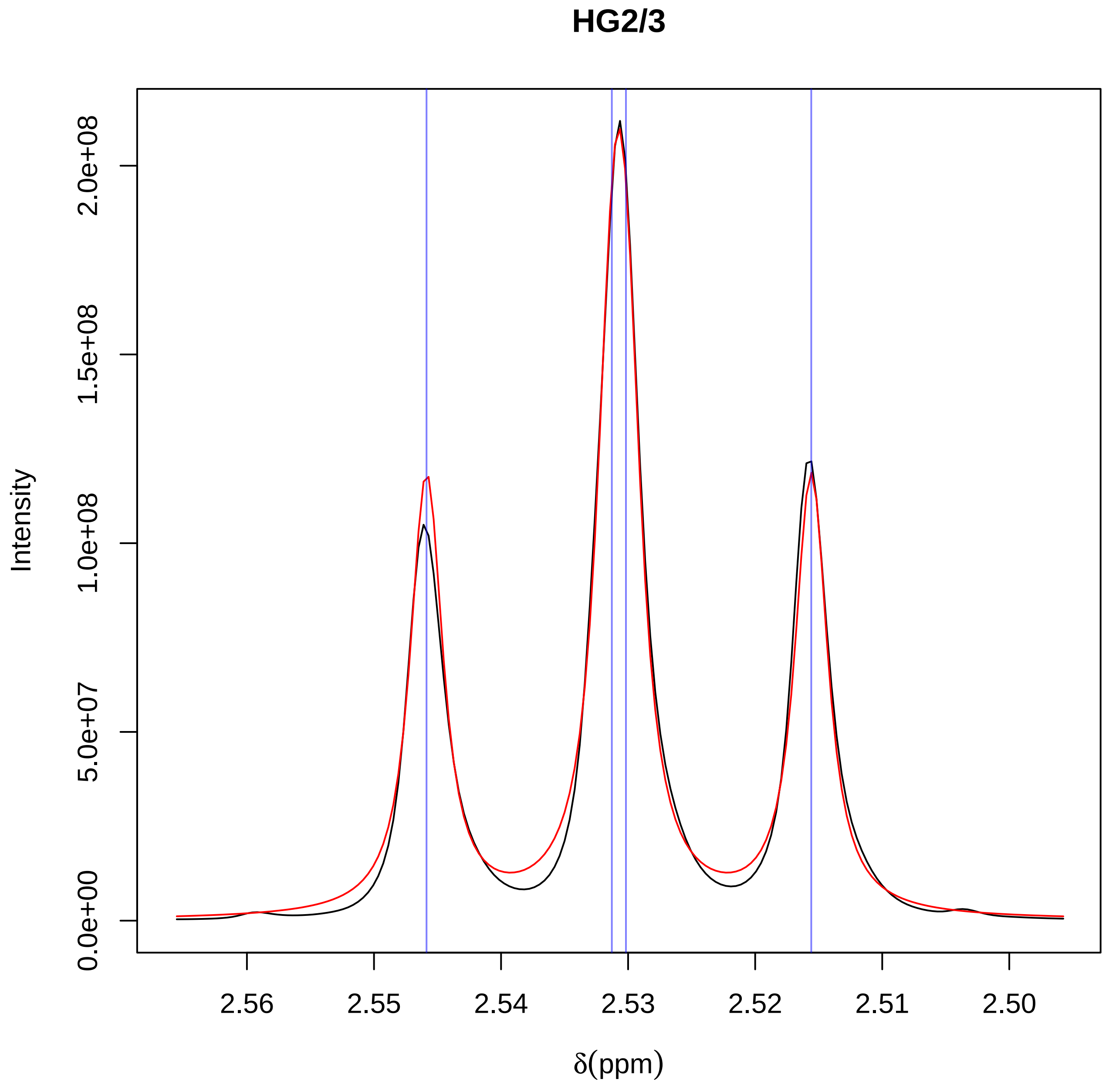

HG2/3
Intensity
0.0e+00
5.0e+07
1.0e+08
1.5e+08
2.0e+08
2.56
2.55
2.54
2.53
2.52
2.51
2.50
δ(ppm)

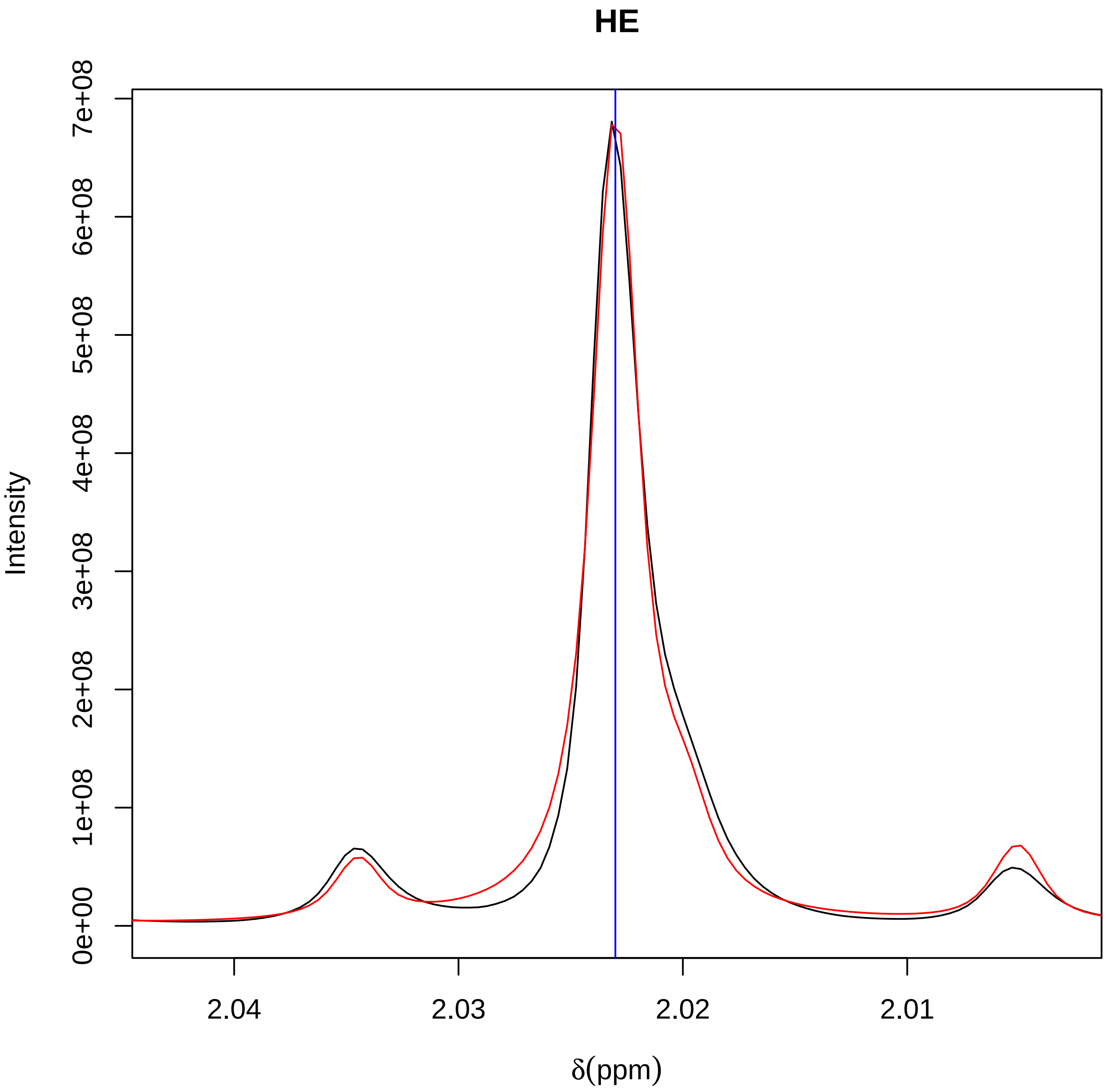

HE
Intensity
0e+00
1e+08
2e+08
3e+08
4e+08
5e+08
6e+08
7e+08
2.04
2.03
2.02
2.01
δ(ppm)
